# Divergent Evolution of PcF/SCR74 Effectors in Oomycetes Is Associated with Distinct Recognition Patterns in Solanaceous Plants

**DOI:** 10.1128/mBio.00947-20

**Published:** 2020-06-30

**Authors:** Xiao Lin, Shumei Wang, Laura de Rond, Nicoletta Bertolin, Roland H. M. Wouters, Doret Wouters, Emmanouil Domazakis, Mulusew Kassa Bitew, Joe Win, Suomeng Dong, Richard G. F. Visser, Paul Birch, Sophien Kamoun, Vivianne G. A. A. Vleeshouwers

**Affiliations:** a Wageningen UR Plant Breeding, Wageningen University and Research, Wageningen, The Netherlands; b Cell and Molecular Sciences, The James Hutton Institute, Dundee, United Kingdom; c The Sainsbury Laboratory, University of East Anglia, Norwich, United Kingdom; d School of Life Sciences, Division of Plant Sciences, University of Dundee at the James Hutton Institute, Dundee, United Kingdom; University of Nebraska-Lincoln

**Keywords:** MAMP, apoplastic effector, surface immune receptor, potato late blight, *Phytophthora infestans*

## Abstract

Plants deploy cell surface receptors known as pattern-recognition receptors (PRRs) that recognize non-self molecules from pathogens and microbes to defend against invaders. PRRs typically recognize microbe-associated molecular patterns (MAMPs) that are usually widely conserved, some even across kingdoms. Here, we report an oomycete-specific family of small secreted cysteine-rich (SCR) proteins that displays divergent patterns of sequence variation in the Irish potato famine pathogen Phytophthora infestans. A subclass that includes the conserved effector PcF from Phytophthora cactorum activates immunity in a wide range of plant species. In contrast, the more diverse SCR74 subclass is specific to *P. infestans* and tends to trigger immune responses only in a limited number of wild potato genotypes. The SCR74 response was recently mapped to a G-type lectin receptor kinase (*G-LecRK*) locus in the wild potato Solanum microdontum subsp. *gigantophyllum.* The *G-LecRK* locus displays a high diversity in *Solanum* host species compared to other solanaceous plants. We propose that the diversification of the SCR74 proteins in *P. infestans* is driven by a fast coevolutionary arms race with cell surface immune receptors in wild potato, which contrasts the presumed slower dynamics between conserved apoplastic effectors and PRRs. Understanding the molecular determinants of plant immune responses to these divergent molecular patterns in oomycetes is expected to contribute to deploying multiple layers of disease resistance in crop plants.

## Abstract (precis)

Immune receptors at the plant cell surface can recognize invading microbes. The perceived microbial molecules are typically widely conserved and therefore the matching surface receptors can detect a broad spectrum of pathogens. Here we describe a family of *Phytophthora* small extracellular proteins that consists of conserved subfamilies that are widely recognized by solanaceous plants. Remarkably, one subclass of SCR74 proteins is highly diverse, restricted to the late blight pathogen *Phytophthora infestans* and is specifically detected in wild potato plants. The diversification of this subfamily exhibits signatures of a coevolutionary arms race with surface receptors in potato. Insights into the molecular interaction between these potato-specific receptors and the recognized *Phytophthora* proteins are expected to contribute to disease resistance breeding in potato.

## INTRODUCTION

The plant apoplast is the battlefront of the plant-pathogen interaction (1). To colonize plants, pathogens secrete an arsenal of apoplastic effector proteins, including small cysteine-rich (SCR) proteins, proteases, and protease inhibitors for facilitating their infection and manipulating the plant immune system (2, 3). Many of these apoplastic pathogen molecules are widely conserved, for example necrosis-inducing proteins (NLPs) that occur in bacteria, fungi, and oomycetes. Microbe-associated molecular patterns (MAMPs), such as flagellin of bacteria, chitin of fungi, and elicitins of oomycetes (4), are typically highly conserved as well, whereas apoplastic effectors that typically represent small SCR proteins exhibit various degrees of conservation, such as AVR2 and AVR4 of Cladosporium fulvum (5). Plants can monitor the extracellular non-self molecules or epitopes and trigger downstream defense responses by deploying surface immune receptors known as pattern recognition receptors (PRRs) that typically consist of receptor-like proteins (RLPs) or receptor-like kinases (RLKs) (6). Most RLPs/RLKs cloned to date, such as flagellin sensing 2 (FLS2), EF-Tu receptor (EFR), and elicitin receptor (ELR), contain an extracellular leucine-rich repeat (LRR) domain (7–9). Recently, various RLKs with other extracellular domains, such as an epidermal growth factor (EGF)-like domain, a LysM domain, or a lectin domain have been found to be involved in plant immunity (6).

In oomycetes, a wide diversity of apoplastic proteins that play a role in modulating host defense responses has been characterized. Most identified apoplastic effectors represent SCR proteins, such as elicitins (10), PcF (*Phytophthora cactorum*-*Fragaria*), SCR74, and SCR91 (11–13). PcF is a 7.67-kDa SCR protein of 73 amino acids, which forms three disulfide bridges by six conserved cysteines, and triggers defense-related responses on strawberry and tomato (11, 14). Additional SCR proteins with a similar domain (the PcF domain; Pfam PF09461) have been further identified, i.e., SCR74 and SCR96, consisting of 74 and 96 amino acids, respectively. *Scr74* belongs to a highly polymorphic gene family that is under positive selection in *P. infestans*. Expression of *Scr74* is significantly upregulated during the early infection stages into host plants (12). Recently, the putative SCR74 receptor gene was fine mapped to a *G-LecRK* locus in wild potato (15). SCR96 is another related protein from *P. cactorum*; however, it lacks the PcF domain. SCR96 triggers cell death responses in some Solanaceae, including Nicotiana benthamiana and tomato (16). So far, very little is known about the function and evolution of these PcF-like effectors, and their targets or receptors in plants are unknown.

In the course of the arms race, effector genes are expected to be the direct target of the evolutionary forces that drive the antagonistic interplay between pathogen and host (17). The evolutionary dynamics of intracellular nucleotide-binding domain and leucine-rich repeat containing (NLR) receptors that mount a hypersensitive response (HR) to host-translocated effectors and delimit pathogen growth are well understood (18, 19). Many plant *NLR* genes are located in highly polymorphic loci and are under strong selection pressure (20). The coevolution of a number of pathogen avirulence (*Avr*) and plant *NLR* genes have been reported to follow the arms race model, such as the *ATR1* from Hyaloperonospora parasitica and *RPP1* from *Arabidopsis* (21), and *AvrL567* in the flax rust fungus Melampsora lini and *L5*, *L6*, and *L7* from flax (22). In contrast, most PRRs are extremely conserved, for example, FLS2 occurs across a wide range of monocotyledonous and dicotyledonous plant species and detects a conserved epitope of bacterial flagellin (7). EFR that recognizes conserved peptides of bacterial EF-Tu is highly conserved within the extensive family of the *Brassicaceae* (8).

*Phytophthora infestans* is a devastating hemi-biotrophic oomycete that causes late blight of potato (23). During early infection phases, hyphae ramify through the intercellular space and form haustoria inside host cells. So far, cytoplasmic effectors of *P. infestans* and the molecular determinants that perceive them have been characterized extensively, but studies on the first line of defense based on apoplastic effectors and their receptors are relatively scarce. Here, we study the PcF/SCR effectors from oomycete plant pathogens by sequence and genome analysis, functional studies *in planta* and we compare the *G-LecRK* loci in different solanaceous genomes. Our findings show that the conserved PcF effector of the PcF/SCR family is widely recognized in solanaceous plant species, whereas SCR74 in *P. infestans* is differentially recognized in wild potato accessions and experiences accelerated evolution rates, potentially in an arms race with a family of *G-LecRK* kinases.

## RESULTS

### PcF/SCR effectors are specific to oomycetes.

To study the PcF/SCR family, 57 PcF domain-containing proteins (PF09461) were obtained from InterPro. The PcF/SCR proteins were only present in oomycetes, including Hyaloperonospora arabidopsidis (2), Phytophthora cactorum (2), Phytophthora capsici (1), Phytophthora parasitica (16), Phytophthora ramorum (1), Phytophthora sojae (4), and Phytophthora infestans (24). Eleven redundant PcF-like proteins were removed, and the remaining 45 PcF/SCR proteins were renamed by the species abbreviation and the number of amino acids of the full-length protein (Table S1 in the supplemental material). Furthermore, by using SCR74 and PcF as the query, we performed tBlastn against 23 public available *Phytophthora* genomes, including P. mirabilis, P. ipomoeae, P. andina, and P. phaseoli, which are close relatives of *P. infestans* (25), and 20 extra PcF/SCR proteins were identified (Table S1). Our data suggest that the PcF/SCR family is restricted to *Peronosporales* and has expanded dramatically in *P. infestans*.

10.1128/mBio.00947-20.8 TABLE S1 List of PcF/SCR proteins and *P. cactorum* isolates used in this study. Download Table S1, XLSX file, 0.02 MB. Copyright © 2020 Lin et al. 2020 Lin et al. This content is distributed under the terms of the Creative Commons Attribution 4.0 International license.

### SCR74 is expanded in *P. infestans*.

**FIG 1:**
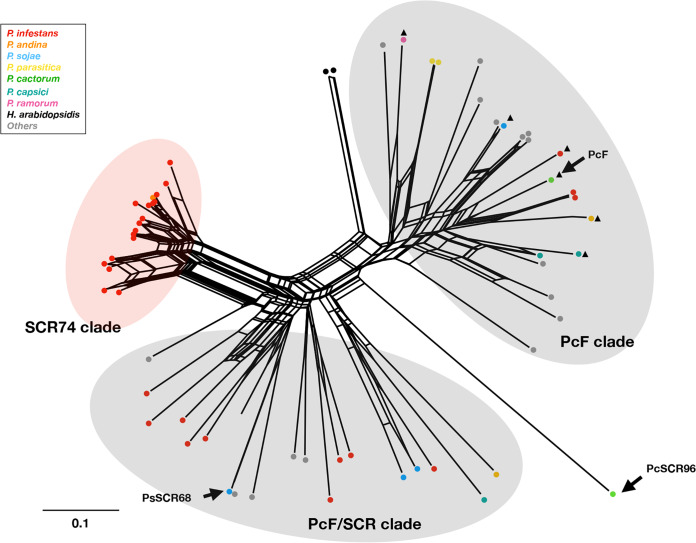
Network of PcF/SCR74 effectors. The network of the 65 PcF/SCR proteins are shown for 19 *Phytophthora* species (including *P. infestans*, *P. andina*, *P. sojae*, *P. parasitica*, *P. cactorum*, *P. capsici*, and *P. ramorum*) (the others are shown in Fig. S1 and Table S1) and *Hyaloperonospora arabidopsidis* (spp. marked by colored dots). The PcF and PcF/SCR74 clades are shaded gray, and the *P. infestans*-specific SCR74 clade is shaded red. PcF orthologs are marked with a black triangle (see Fig. 2A).

To analyze the sequence diversity and phylogeny of the PcF/SCR family, the PcF domains of the 65 PcF/SCR proteins were subjected to sequence alignment by MAFFT and a NJ tree was generated (Fig. S1). Due to reticulate sequence exchange events that might have happened in this family (12), a network analysis was also made to reflect the phylogeny (Fig. 1). PcSCR96 from *P. cactorum* was included as an outgroup. Based on the alignment and network analysis, the PcF/SCR proteins were classified into three clades, i.e., a PcF clade, an SCR74 clade, and a PcF/SCR clade, respectively (Fig. 1, Fig. S1). All full-length PcF/SCR proteins from *Phytophthora* contain 6 to 8 highly conserved cysteines that are involved in S-bridge formation, and a conserved motif, Y/HSxS/ANXXI/VSQ/K of 18 to 27 amino acids (aa). A highly variable region from amino acid position 31 to 51 is present in these PcF/SCR proteins; members of the SCR74 clade share an AINA/PD/EPV/IA motif, that is different in the other clades (Fig. S1). Of note, this SCR74 clade consists only of variants from *P. infestans*, and 1 SCR74 protein from *P. andina*, which is a hybrid of *P. infestans* (26). In contrast, the PcF clade and the PcF/SCR clade contains proteins from various species. Overall, the PcF/SCR family occurs as three clades, from which the SCR74 clade seems to have evolved specifically in *P. infestans* (Fig. 1).

10.1128/mBio.00947-20.1 FIG S1 Alignment of the PcF domain (PF09461) of 65 PcF/SCR proteins. The 65 PcF/SCR proteins are classified into 3 clades, i.e., a PcF clade, a PcF/SCR clade, and an SCR74 clade. PcSCR96 was used as outgroup for phylogeny analysis. Download FIG S1, TIF file, 2.4 MB. Copyright © 2020 Lin et al. 2020 Lin et al. This content is distributed under the terms of the Creative Commons Attribution 4.0 International license.

### PcF is a conserved apoplastic effector of *Phytophthora*.

**FIG 2:**
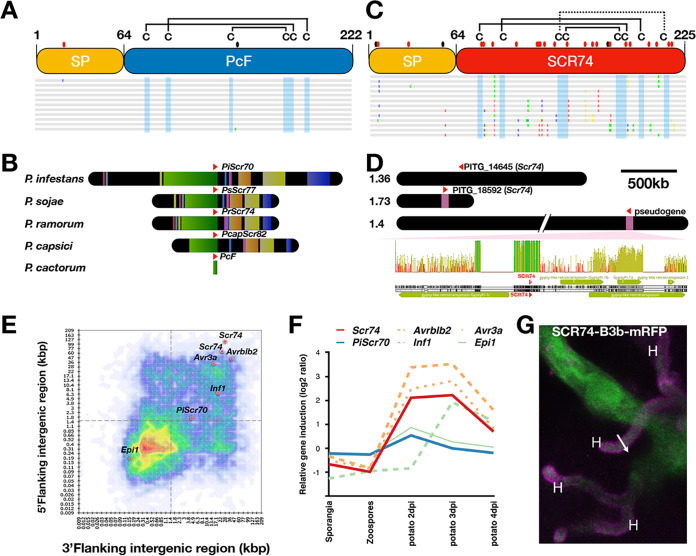
PcF and Scr74 possess MAMP and effector characteristics, respectively. (A) Graphical representation of a sequence alignment of *PcF* genes from nine *P. cactorum* isolates from the USA and Europe (Table S1). The polymorphic amino acids are highlighted by different colors in the alignment, the synonymous and nonsynonymous SNP are shown by black and red dots, respectively. The cysteine residues are shaded by blue, the cysteines and the predicted disulfide bridge are marked by black lines. (B) *PcF* flanking sequences from *P. infestans* (500 kb), *P. sojae*, *P. ramorum*, *P. capsici* (200 kb), and *P. cactorum* (a short contig) containing the *PcF* orthologs *PiScr70*, *PsScr77*, *PrScr74*, *PcapScr82*, and *PcF* (red arrows), respectively, were aligned by Mauve. Regions of significant synteny are displayed as colored locally colinear blocks (LCBs) based on Mauve’s progressive algorithm. (C) Graphical representation of a DNA sequence alignment of 13 *Scr74* variants from *P. infestans*. The predicted polymorphic amino acids are highlighted by different colors in the alignment, the synonymous and nonsynonymous SNP are shown by black and red dots above the illustration, respectively. The predicted cysteines are shaded blue, and the disulfide bridges are marked by black lines. (D) The *Scr74* homologs (red arrows) *PITG_14645*, *PITG_18592*, and a pseudogene originate from supercontigs 1.36, 1.73, and 1.4, respectively. Regions (15 kb) from supercontig 1.73 and supercontig 1.4 were extracted for alignment. The pairwise identity is illustrated by the bars above the sequence alignment (100%, green; 30 to 100%, yellow; <30%, red). The *Scr74* genes and the flanking 3 kb show synteny in these two supercontigs. The gypsy retrotransposons are annotated by green arrows. (E) The distance between flanking genes of the reference *P. infestans* isolate T30-4 were plotted in a heatmap, where the *x* and *y* axes present the 3′ and 5′ intergenic distances, respectively. The gene density is shown by different colors. The intergenic gene distances of *Epi1* (*PITG_22681*), *PiScr70* (*PITG_22677*), *Avr3a* (*PITG_14371*), and *Avrblb2* (*PITG_20300*), as well as two *Scr74* homologs (*PITG_14645* and *PITG_18592*), are plotted on the heatmap. (F) The relative expression pattern of *Avr3a* (*PITG_14371*), *Inf1* (*PITG_12551*), *Epi1* (*PITG_22681*), *Avrblb2* (*PITG_20300*), and *PiSCR70* (*PITG_22677*) in different structures and infection stages, including sporangia, zoospores, and 2, 3, and 4 days after inoculation on potato. (G) Confocal projections reveal that SCR74-B3b-mRFP fusion proteins of *P. infestans* transformants are secreted at haustoria (H) during infection of Nicotiana benthamiana. GFP was imaged with 488 nm excitation and emissions collected between 500 and 530 nm, respectively. mRFP fluorescent proteins were excited with 561 nm light and fluorophore emission was detected between 600 and 630 nm. Projections were collected from leaf tissue infected by *P. infestans* transformants.

So far, two PcF variants from *P. cactorum* were reported (11, 16). To study the sequence polymorphism that occurs for *PcF* genes, *PcF* orthologs from nine *P. cactorum* strains, isolated from the United States or Europe, were amplified and sequenced. The sequence alignments indicate that *PcF* genes are highly conserved in all tested *P. cactorum* isolates (Fig. 2A). Only one nonsynonymous mutation was found in the predicted signal peptide of PcF (PcF-AF354650), and another synonymous mutation was found in the effector domain of NL2003-3 (Fig. 2A, Fig. S2). The amino acid sequence of the effector domain was fully conserved for all identified *PcF* homologs. Our results indicate that *PcF* genes are highly conserved and appear to undergo purifying selection in *P. cactorum* strains from different geographic locations.

10.1128/mBio.00947-20.2 FIG S2 *PcF* loci are conserved in different oomycetes and *PcF* genes are conserved in different P. cactorum isolates. (A) Amino acid alignment of PcF proteins from nine *P. cactorum* isolates from the USA and Europe. (B) Flanking sequences of 500 kb of PcF from P. infestans and 200 kb of PcF from P. sojae, P. ramorum, and P. capsici, along with a short contig from *P. cactorum*, were aligned by Mauve. Regions of significant synteny are displayed as colored locally collinear blocks (LCBs) based on Mauve’s progressive algorithm. The LCBs are connected by colored lines between the species. The *PcF* orthologs (*PiSCR70*, *PsSCR77*, *PrSCR74*, *PcapSCR82*, and *PcF*) from the 5 *Phytophthora* species are shown with black arrows. Download FIG S2, TIF file, 1.7 MB. Copyright © 2020 Lin et al. 2020 Lin et al. This content is distributed under the terms of the Creative Commons Attribution 4.0 International license.

To further study whether *PcF* loci are conserved in diverse *Phytophthora* species, we extracted the *PcF* loci and the flanking 250-kb region from the genome of *P. infestans*, the 100-kb flanking sequence from P. sojae, P. ramorum, and P. capsici, and a short contig containing *PcF* from *P. cactorum.* Sequence alignment of the *PcF* loci (Fig. 2B, Fig. S2) shows a colinear structure of *PcF* loci in *Phytophthora*. Considering that these *Phytophthora* species cover the breadth of diversity of the genus, we postulate that *PcF* is an ancient and fairly conserved gene in *Phytophthora*.

### *Scr74* is a fast-evolving apoplastic effector.

SCR74 proteins were reported to be highly diverse and under strong positive selection pressure, based on 21 *scr74* variants from 8 *P. infestans* strains (12) (Fig. 2C). With the increased amount of NGS data, we reevaluated the sequence diversity of SCR74 for 52 *P. infestans* isolates present in the public databases and two *P. infestans* isolates sequenced in this study (Fig. S3). Our observation supports the previous findings, that: (i) *Scr74* genes are present in all sequenced *P. infestans* isolates; (ii) the sequences of *Scr74* genes are highly diverse and display a marked signature of positive selection as previously reported by Liu et al. (12); and (iii) the cysteine residues are conserved in all tested SCR74 proteins.

10.1128/mBio.00947-20.3 FIG S3 Polymorphisms of SCR74 genes from 52 sequenced *P. infestans* isolates. The sequencing reads from 52 *P. infestans* isolates were mapped to the SCR74-B3b sequence, and the SNPs are shown as black dots. The protein consists of a 21-amino-acid signal peptide (SP, blue bar), and a 53-amino-acid mature protein (black bar). The cysteine residues are highlighted in yellow and they are conserved in most of the variants. Other conserved amino acids with no change or only synonymous mutation are highlighted in blue. The highly diverse amino acids with nonsynonymous mutations are highlighted in red. Download FIG S3, TIF file, 2.6 MB. Copyright © 2020 Lin et al. 2020 Lin et al. This content is distributed under the terms of the Creative Commons Attribution 4.0 International license.

To study the genomic architecture of *Scr74* genes in the *P. infestans* reference genome, we extracted three *Scr74*-containing supercontigs (1.36, 1.73 and 1.4) (Fig. 2D) from the *P. infestans* reference genome. There are three *Scr74* homologs, including a pseudogene in supercontig 1.4. By comparing the flanking region of these *Scr74* loci, we found the *Scr74* genes and the flanking regions (∼2 kb) from supercontigs 1.73 and 1.4 showed a high level of identity (Fig. 2D). This observation points to a translocation event at the *Scr74* loci, which might have been driven by gypsy transposons surrounding these *Scr74* genes.

Most oomycete genomes have gene-dense housekeeping regions (GDRs) and gene-sparse repeat-rich regions (GSRs), and rapidly evolving effectors tend to be located in the GSR (27). To visualize whether *Scr74* and *PcF* localize in GSR or gene-dense regions (GDRs), we plotted two *Scr74* genes and *PiScr70*, the *PcF* ortholog of *P. infestans*, as well as known apoplastic effectors *Inf1* and *Epi1*, and the well-characterized cytoplasmic *Avrblb2* and *Avr3a* on the flanking intergenic regions (FIRs) map of *P. infestans* reference genome (T30-4). The *Scr74* genes localize to the extreme GSR region, similar to the *Avr* genes *Avrblb2* and *Avr3a*. In contrast, the *P. infestans PcF* ortholog *PiScr70* lands closer to the GDR, similar to *Inf1*, which shares features with MAMPs (28) (Fig. 2E). Additionally, to study the expression profile of selected apoplastic and cytoplasmic effectors, cDNA microarray data of *P. infestans* reference isolate T30-4-infected samples were plotted for various stages (29). We found the expression of *Scr74* genes peaked at 2 to 3 days after infection (dpi), which is similar to typical *Avr* genes, whereas the expression pattern of *PiScr70* rather resembles Epi1 (Fig. 2F).

To investigate the localization of SCR74-B3b *in planta*, *P. infestans* transformants were generated that constitutively express free green fluorescent protein (GFP) in the cytoplasm, and stably expressed either SCR74-B3b or a cysteine mutant SCR74-27A, both with monomeric red fluorescent protein (mRFP) under the control of the constitutive Ham34 promoter (Fig. S4). The transformed *P. infestans* strains were spot-inoculated on N. benthamiana leaves. Confocal microscopy revealed that SCR74-B3b-mRFP proteins clearly accumulate at haustoria (Fig. 2G, Fig. S4), indicating that haustoria are the main secretion sites for SCR74, as also reported for *Avr* genes (30, 31).

10.1128/mBio.00947-20.4 FIG S4 *Phytophthora infestans* apoplastic effector SCR74-B3b is secreted at haustoria. (A) The expression of SCR74-B3b-mRFP and a cysteine mutant SCR74-B3b-27A-mRFP were confirmed in mycelium (M) and culture filtrate (CF) using immunoblotting with αmRFP antibody, and αGFP primary antibody was used to detect intercellular protein GFP to show there was no leakage in the CF with cellular proteins. Ponceau stain (PS) was used for protein loading control. Protein size markers are indicated in kDa. (B) Confocal projections reveal that both fusion proteins of SCR74-B3b-mRFP and SCR74-B3b-27A-mRFP are secreted at haustoria (H) in infected tissues by *P. infestans* transformants expressing SCR74-B3b-mRFP and SCR74-B3b-27A-mRFP, respectively. Download FIG S4, TIF file, 2.3 MB. Copyright © 2020 Lin et al. 2020 Lin et al. This content is distributed under the terms of the Creative Commons Attribution 4.0 International license.

### PcF and SCR74 exhibit different recognition patterns.

**FIG 3:**
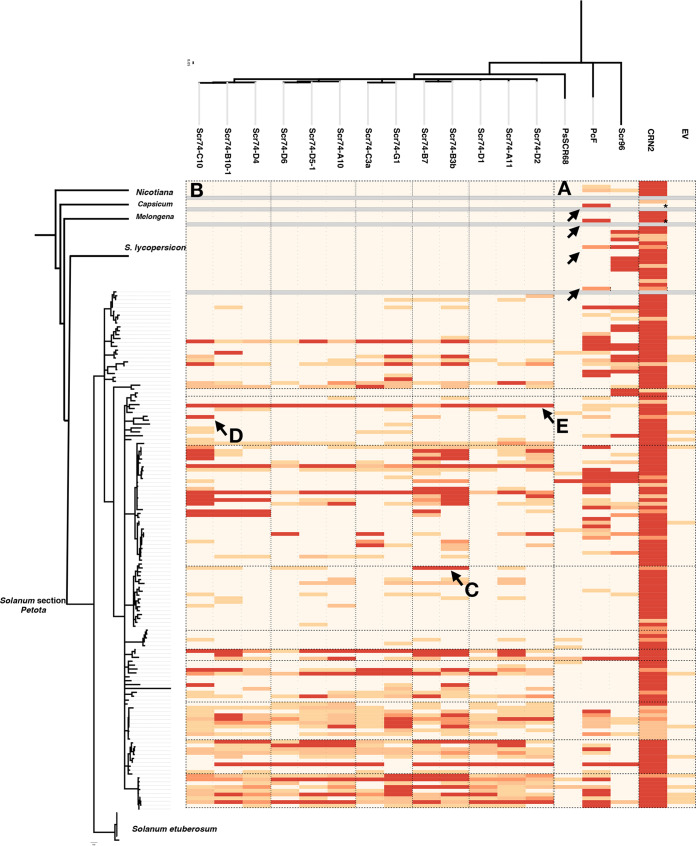
Effectoromics screening of PcF/SCR effectors on plants of the *Solanaceae*. The intensity of cell-death response after PVX agro-infection of apoplastic effectors in leaves is represented by a heat map that ranges from dark red (strong response, average score >8), dark orange (score 7 to 8), light orange (score 5 to 6), to beige (score 0 to 4). CRN2 and the empty pGR106 vector were used as positive and negative controls, respectively. The asterisks highlight a pepper and an eggplant accession that failed to respond to CRN2-pGR106, however, PcF response were reproducible in three independent agro-infiltration experiments with coinfiltration of R3b and Avr3b as positive controls. A Bayesian tree of *Solanum* section *Petota* was generated based on previously produced AFLP data, and S. etuberosum genotypes were used as outgroup (45). The phylogeny of other *Solanaceae* species is the illustration based on classical taxonomy (46). For the PcF/SCR effectors, a NJ tree was made based on the PcF domain, and PcSCR96 was used as outgroup. The gray blocks represent spacers between plant clades. (A) Widespread recognition of PcF and SCR96 in various *Solanaceae*. (B) Similar recognition pattern of SCR74-C10, SCR74-B10-1, and SCR74-D4 in various wild potato species. (C) Specific response to SCR74-B3b and SCR74-B7 in *Solanum microdontum* subsp. *gigantophyllum* GIG362-6. (D) Highly restricted response to SCR74-C10 in *Solanum stoloniferum* STO389-4 (Table S2). (E) Broad response to all SCR74 variants in *Solanum chacoense* CHC338-1.

To bridge the sequence analysis with the function of these PcF/SCR proteins, we performed an effectoromics screening in a wide range of solanaceous plants. We tested 245 genotypes, which included 206 wild tuber-bearing potato (*Solanum* section *Petota*), 23 tomato, 7 eggplant, 10 pepper, and 8 *Nicotiana* genotypes. *PcF* and SCR96 from *P. cactorum*, SCR68 from *P. sojae*, and 13 SCR74 variants from *P. infestans* were cloned into potato virus X (PVX) vectors pGWC-PVX or pGR106, and transformed into Agrobacterium tumefaciens strain GV3101 for transient expression. The *Agrobacterium* clones carrying single PcF/SCR genes were toothpick-inoculated onto at least 6 leaves from 3 plants. The general necrosis-inducing CRN2 and the empty vector were used as positive and negative controls, respectively. The symptoms were scored 12 to 14 days after infection, on a range of 0 to 10, reflecting no visible response up to clear cell death in all replicates, respectively. After removing genotypes that showed unspecific cell death to pGR106 treatment, or failed to show cell death to pGR106-CRN2, there were a total of 4 *Nicotiana*, 2 pepper, 3 eggplant, 17 tomato, and 136 potato genotypes that were scored for their response to the effectors (Fig. 3, Table S2).

10.1128/mBio.00947-20.9 TABLE S2 Effectoromics screening on *Solanaceae* genotypes. The intensity of cell-death response after PVX agro-infection of apoplastic effectors in leaves is represented by a heat map that ranges from dark red (strong response, average score >8), dark orange (score 7 to 8), light orange (score 5 to 6), to beige (score 0 to 4). CRN2 and empty pGR106 vector were used as positive and negative controls, respectively. Download Table S2, XLSX file, 0.03 MB. Copyright © 2020 Lin et al. 2020 Lin et al. This content is distributed under the terms of the Creative Commons Attribution 4.0 International license.

Our effectoromics screens showed that PcF and SCR96 from *P. cactorum* caused cell death responses in a wide range of diverse *Solanaceae*. Recognition was detected in various wild potato species, as well as tomato, pepper, eggplant, and some tobacco accessions (Fig. 3A, Fig. S5). In contrast, recognition of the *P. infestans-*specific effector SCR74 was restricted to *Solanum* section *Petota* and no response was noted in any other Solanaceous plants (Fig. 3B). The pattern of responses to SCR74 variants was highly specific, but did not seem to show any correlation to clade, species, or geographic origin. For example, most genotypes from *Solanum microdontum* and *Solanum microdontum* subsp. *gigantophyllum* did not recognize any of the tested SCR74 variants, but GIG362-6 showed very clear responses to SCR74-B3b and SCR74-B7 (Fig. 3C, Fig. S5). In contrast, some genotypes, such as Solanum chacoense CHC338-1 (Fig. 3E), showed response to all tested SCR74 variants, as well as PcF and SCR96, but not to SCR68. SCR68 failed to cause cell death in most tested plants, and we only detected a specific response in S. stoloniferum STO389-4 (Table S2). Collectively, our functional screening indicates that the recognition of the conserved PcF effector is widespread in the *Solanaceae*, whereas recognition of the highly diverse, *P. infestans*-specific SCR74 is restricted to tuber-bearing potato accessions.

10.1128/mBio.00947-20.5 FIG S5 PcF and SCR74 responsiveness in the *Solanaceae*. (A) PVX agro-infection of PcF in Capsicum annuum (CGN16796), Solanum incanum (CGN18575), Solanum hjertingii (HJT350-1), and Solanum polytrichon (PLT789-6), and agro-infiltration in Solanum lycopersicum (CGN14330). (B and C) PVX agro-infection of SCR74 variants on *S. polytrichon* (PLT378-1) (B) and Solanum microdontum subsp. *gigantophyllum* (GIG362-6) (C). CRN2 is included as a positive control for PVX agro-infection, and coinfiltration of R3b and Avr3b for agro-infiltration. The empty PVX vector is used as negative control. Download FIG S5, TIF file, 2.4 MB. Copyright © 2020 Lin et al. 2020 Lin et al. This content is distributed under the terms of the Creative Commons Attribution 4.0 International license.

To further explore the specificity of SCR74 recognition in wild potato, we compared the responses of the potato genotypes with the phylogenic relationships of the SCR74 members. For all individual SCR74 variants, at least one responding wild potato was identified and patterns of recognition were discerned. We noted that SCR74 variants that were classified in a same cluster, such as SCR74-C10, -B10-1, and -D4 (Fig. 3D, Fig. S1) were in many cases causing cell death in the same set of genotypes, apart from exceptions such as PLT378-2 (Fig. S6). Similarly, examples such as SCR74-B3b and SCR74-B7, which only differ in two polymorphic amino acids (Fig. S1), share specific cell death profiles of some sets of *Solanum* genotypes (Fig. 3C, Table S2). These results indicate that multiple SCR74 receptors are present and that they recognize different but closely related SCR74 variants.

10.1128/mBio.00947-20.6 FIG S6 Single amino acid change of SCR74 leads to altered recognition specificity. (A) Protein alignment of SCR74-D4, -B4, and -C10 The predicted S-S bridges (yellow bars) and the α-helix (green bars) are indicated. (B) Predicted structure of the mature SCR74 protein, where the polymorphic amino acid between SCR74-D4, - B4, and -C10 at position 28 is shown. (C) PTA767-1 recognizes SCR74-D4, -B4, and -C10, whereas PLT378-2 can only recognize SCR74-C10. (D) Two SCR74 cysteine mutations SCR74-synB3b-27A and SCR74-synB3b-47A were synthesized and PVX agro-infected on GIG362-6 leaves together with SCR74-B3b and a codon-optimized SCR74-B3b. CRN2 and empty vector were used as positive and negative controls, respectively. The photo was taken 14 dpi. Download FIG S6, TIF file, 2.2 MB. Copyright © 2020 Lin et al. 2020 Lin et al. This content is distributed under the terms of the Creative Commons Attribution 4.0 International license.

To test if the cysteines are important for the SCR74 activity, we synthesized two SCR74-B3b cysteine mutants and functionally tested them in SCR74-responding *Solanum microdontum* subsp. *gigantophyllum* genotype GIG362-6 plants. The mutants failed to cause cell death, showing that S-bridges are critical for SCR74 function (Fig. S6D).

### *G-LecRK* locus in wild potato mediates the response to SCR74-B3b.

**FIG 4:**
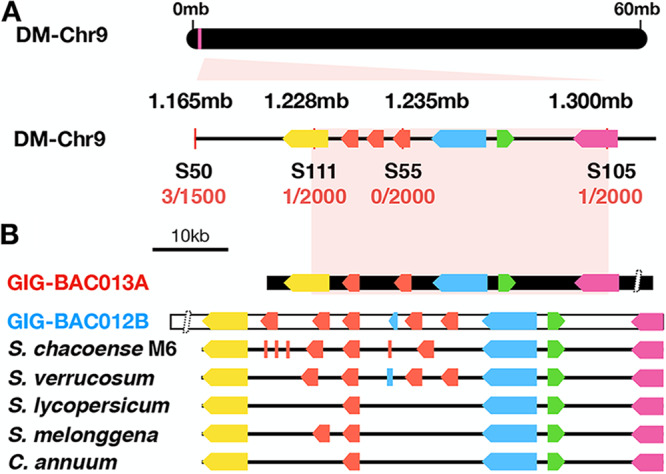
The candidate SCR74 receptor is located on a highly diverse *G-LecRK* locus. (A) The candidate SCR74-B3b receptor is located in a 73-kb (0.1cM) region between marker S111 and S105 on chromosome 9, based on the reference clone DM1-3 genome, and 43-kb on GIG-BAC013A. The numbers of recombination events are shown in red (15). (B) Two BAC clones GIG-BAC013A and GIG-BAC012B from GIG362-6 were isolated and sequenced. GIG-BAC013A (red) represents the haplotype with the candidate SCR74 receptor. GIG-BAC012B (blue) represents another haplotype from GIG362-6. The genomic region of pepper (C. annuum), eggplant (S. melongena), tomato (S. lycopersicum), S. verrucosum, S. chacoense M6, and of 2 haploytypes from *S. microdontum* subsp. *gigantophyllum* (genotype GIG362-6) are shown. Predicted genes are represented as arrows, i.e., *G-LecRK* (red), putative reticulata related 1-like genes (yellow), ATG1c-like genes (blue), prenylated rab acceptor family (green), and uracil phosphoribosyltransfease genes (pink).

Recently, with a newly developed RLP/RLK gene enrichment sequencing (RLP/KSeq), we mapped the response to SCR74 to a locus at the top of chromosome 9 in GIG362-6 (15). Based on the reference genome *S. tuberosum* Group *Phureja* clone DM1-3, the mapping interval contains eight genes, i.e., three receptor-like kinases with a G-type lectin domain (*G-LecRK*) genes, a putative reticulate-related 1 like gene, a serine/threonine-protein kinase ATG1c-like (autophagy-related protein) gene, a prenylated rab acceptor family gene, and a uracil phosphoribosyltransferase-encoding gene (Fig. 4C, Fig. S7). Previously, we had isolated the BAC clone on the responsiveness haplotype of GIG362-6 (15), here we isolated the BAC clone from another haplotype of GIG362-6, and, strikingly, two and five *G-LecRK* genes were found in the responsive and nonresponsive haplotypes, respectively (Fig. 4B).

10.1128/mBio.00947-20.7 FIG S7 Differential expression of candidate genes on haplotype 1 of GIG362-6. (A) The mapping interval on GIG362-6 with the RNA-seq reads from GIG362-6, mapped to haplotype 1 (BAC03-H3 and BAC01-3A). The blue peaks present the coverage of RNA-seq reads. The samples of water treatment and UK3928A inoculation are shown. The two *G-LecRKs* are upregulated after UK3928A infection. (B) Differential expression of candidate genes in the mapping interval. The raw read counts and transcript per million (TPM) for each sample, as well as the differential expression ratio and the *P* value, are shown for the four treatments of GIG_water versus GIG_UK3928A and MCD_UK3928A versus GIG_UK3928A. The upregulated genes are highlighted in red, the downregulated genes are highlighted in blue. The significant (*P* < 0.05) differential expression *P* values are shown in red font, otherwise in blue font. Download FIG S7, TIF file, 2.3 MB. Copyright © 2020 Lin et al. 2020 Lin et al. This content is distributed under the terms of the Creative Commons Attribution 4.0 International license.

To investigate whether the *G-LecRK* loci are conserved among different *Solanaceae*, we analyzed the *G-LecRK* loci from various other available solanaceous genomes. We found that *Solanum chacoense*, which is closely related to *S. microdontum* and clone DM1-3, contains four partial and three full-length *G-LecRK* genes in the locus. In another wild potato, Solanum verrucosum, we detected four *G-LecRK* genes (24). The more distantly related pepper and tomato isolates contained only one *G-LecRK*, and eggplant contained two (Fig. 4B). The copy number variation (CNV) data indicate that the *G-LecRK* loci are highly diverse and they seem expanded in wild potato species.

To evaluate the gene expression level of the candidate genes during *P. infestans* infection, we performed a transcriptome sequencing (RNA-seq) experiment on the mapping parents GIG362-6 and MCD360-1, 48 h postinoculation (hpi) with *P. infestans* isolate UK3928A or mock-inoculated with water. The RNA-seq reads were mapped to the BAC sequences of GIG362-6 and show that the *G-LecRK* genes are upregulated after infection (Fig. S7), which suggests they may play role in the interaction with *P. infestans*.

## DISCUSSION

Plants and pathogens undergo an endless coevolutionary tug of war. Until now, the far majority of molecular studies have focused on cytoplasmic effectors representing *Avr* genes that coevolve with plant NLR receptors (32). However, the degree of coevolution between surface immune receptors and apoplastic effectors has been understudied. Traditionally, many apoplastic effectors were thought to be conserved, MAMP-like molecules. However, the boundary between the MAMPs and effectors, and consequently between MAMP-triggered immunity (MTI) and effector-triggered immunity (ETI), is less strict in many cases. The invasion model describes recognition between those ligand/receptor molecules as a process that continuously takes place during host infection (33, 34). In this study, we build further on the invasion model and show that subclades of an apoplastic effector family in oomycetes have undergone divergent evolutionary paths.

A family of PcF/SCR74 effectors that share a PcF domain occurs in *Peronosporales*, and four subclades can be distinguished (11). We found that the subclade of *PcF* is conserved in *Phytophthora* species, as *PcF* orthologs share a high sequence identity and a colinear structure among various *Phytophthora* genomes. Similar to typical MAMPs, such as flagellin, PcF is widely recognized by diverse plant species, like pepper, eggplant, tomato, and potato, and recognition even occurs beyond the *Solanaceae*, e.g., strawberry (11). In contrast, SCR74 variants are exclusively present in *P. infestans*, with their sequences highly diverse and under strong positive selection pressure (12). We found the recognition of SCR74 variants is restricted to wild potato host plants. Therefore, we conclude that although PcF and SCR74 belong to the same effector family, they are shaped under a divergent evolutionary path during coevolution with their host. PcF/SCR74 clades 1 and 2 represent intermediates, leading to blurred boundaries between typical MAMPs and effectors (34). Our findings suggest that the apoplastic (SCR74) effectors likely evolved from the conserved PcF molecules and underwent a coevolutionary arms race in the host species of *P. infestans*.

The gene conferring response to SCR74 has been fine mapped to a locus of *G-lecRK* that shows upregulation upon *P. infestans* infection (15), which suggests that these *G-LecRK* genes are the most likely candidates for encoding the SCR74 receptor. A few other *G-LecRK* genes have recently been reported to be involved in plant immunity, e.g: *I-3* from tomato conferring resistance to Fusarium oxysporum. Also for *I-3*, functional complementation of the candidate *G-LecRK* gene has not been achieved yet, perhaps because some surfaces receptors often act in networks and require multiple components (35). Other *G-LecRK* examples are *Pi-d2* and *OsLecRK1-3*, conferring resistance to Magnaporthe oryzae and brown planthopper, respectively, and *LORE* from *Arabidopsis* that can mediate bacterial lipopolysaccharide-copurified medium-chain 3-hydroxy fatty acid (mc-3-OH-FA) sensing (24, 32–35). Additionally, *SRK*, a well-characterized *G-LecRK* from *Brassica* is the female determinant of self-incompatibility (SI) (36) that recognizes the S-haplotype-specific SCR/SP11 from self-pollen (36, 37). This points to remarkable parallels between plant immunity and SI as a “social disease,” where both systems include the invading of a host cell by a tubular cell; both interactions are driven by highly diverse G-LecRK receptors and SCR ligands; and both outcomes of the incompatible responses lead to cell death (38).

The *G-LecRK* genes show CNV in the two haplotypes of GIG362-6, with two or five copies, respectively. The copy number of these *G-LecRKs* in different potato genomes varies dramatically, namely, three, four and seven full-length or partial *G-LecRK* genes were found in the DM1-3 potato, *Solanum verrucosum*, and *Solanum chacoense* genomes, respectively, which suggests this locus has been under evolutionary pressure in wild potato species. Other, more distant *Solanaceae*, such as tomato, pepper, and eggplant, only contained one or a maximum of two *G-LecRK* genes in their genome. Our genetic data provide further evidence about the coevolution hypothesis that the highly diverse apoplastic SCR74 effectors coevolve with the receptors in their wild potato host species.

This study contributes to deeper insight into the molecular dialogue between oomycetes and their hosts, in particular for *P. infestans* and potato. We showed that the PcF/SCR effector family acts as “invasion patterns” (33, 34) that have experienced distinct evolutionary trajectories during coevolution with their host. This work also has implications for breeding sustainable resistance to *P. infestans*. To date, breeding for resistance against late blight has had an emphasis on the *NLR* genes, which are typically defeated rapidly by the fast-evolving and highly adaptable *P. infestans*. The *G-LecRK* locus we identified as mediating response to SCR74-B3b is a new source of immune receptors from wild potatoes that complements other recently discovered PRRs that operate against *P. infestans* (9, 39). Stacking these surface immune receptors and combining them with NLRs might provide a tool to target a wide spectrum of the *P. infestans* population and contribute a new source of disease resistance into potato breeding.

## MATERIALS AND METHODS

### Phylogenetic analysis of PcF/SCR proteins.

PcF domain-containing proteins (IPR018570) were obtained from InterPro. The protein sequences were aligned by MAFFT v7.309 (40) and Geneious R10. Redundant sequences were removed manually based on the alignment outputs. A neighbor-joining tree was performed by Geneious R10, using the Jukes-Cantor model. The phylogeny network was made by SplitTree4 (41). More details are in the Materials and Methods section of the supplemental materials.

### Genome data and sequence analysis.

The oomycete genomes were obtained from EnsemblProtists (http://protists.ensembl.org/) or JGI genome portal (https://genome.jgi.doe.gov), including *P. infestans* (ASM14294v1) (29), *P. sojae* (*P. sojae* V3.0), *P. ramorum* (ASM14973v1) (42), and *P. capsici* (LT1534 v11.0) (43). The draft genome of *P. cactorum* strain LV007 can be obtained from GenBank (NBIJ01000000) (44). More details are in the Materials and Methods section of the supplemental materials.

***Phytophthora* isolates.** *Phytophthora cactorum* isolates that were used in this study are listed in Table S1.

### Plant material.

The seeds of tomato, pepper, and eggplant were obtained from the Centre for Genetic Resources, Wageningen, The Netherlands (CGN). The potato genotypes were clonally maintained at the *in vitro Solanum* collection of Plant Breeding at Wageningen University and Research.

### PVX agro-infection and agro-infiltration in plants.

The effectors were cloned into pGR106 vector and then transformed into Agrobacterium tumefaciens strain GV3101 for PVX agro-infection or into pK7WG2 for agro-infiltration. More details are in the Materials and Methods section of the supplemental materials.

10.1128/mBio.00947-20.10 TEXT S1 Detailed Materials and Methods used in this study. Download Text S1, DOCX file, 0.04 MB. Copyright © 2020 Lin et al. 2020 Lin et al. This content is distributed under the terms of the Creative Commons Attribution 4.0 International license.
