# Supplementary figures and images for: Divergent Evolution of PcF/SCR74 Effectors in Oomycetes Is Associated with Distinct Recognition Patterns in Solanaceous Plants
Source: mBio. 2020 Jun 30;11(3):e00947-20. doi: 10.1128/mBio.00947-20 (PMC7327169; doi:10.1128/mBio.00947-20)

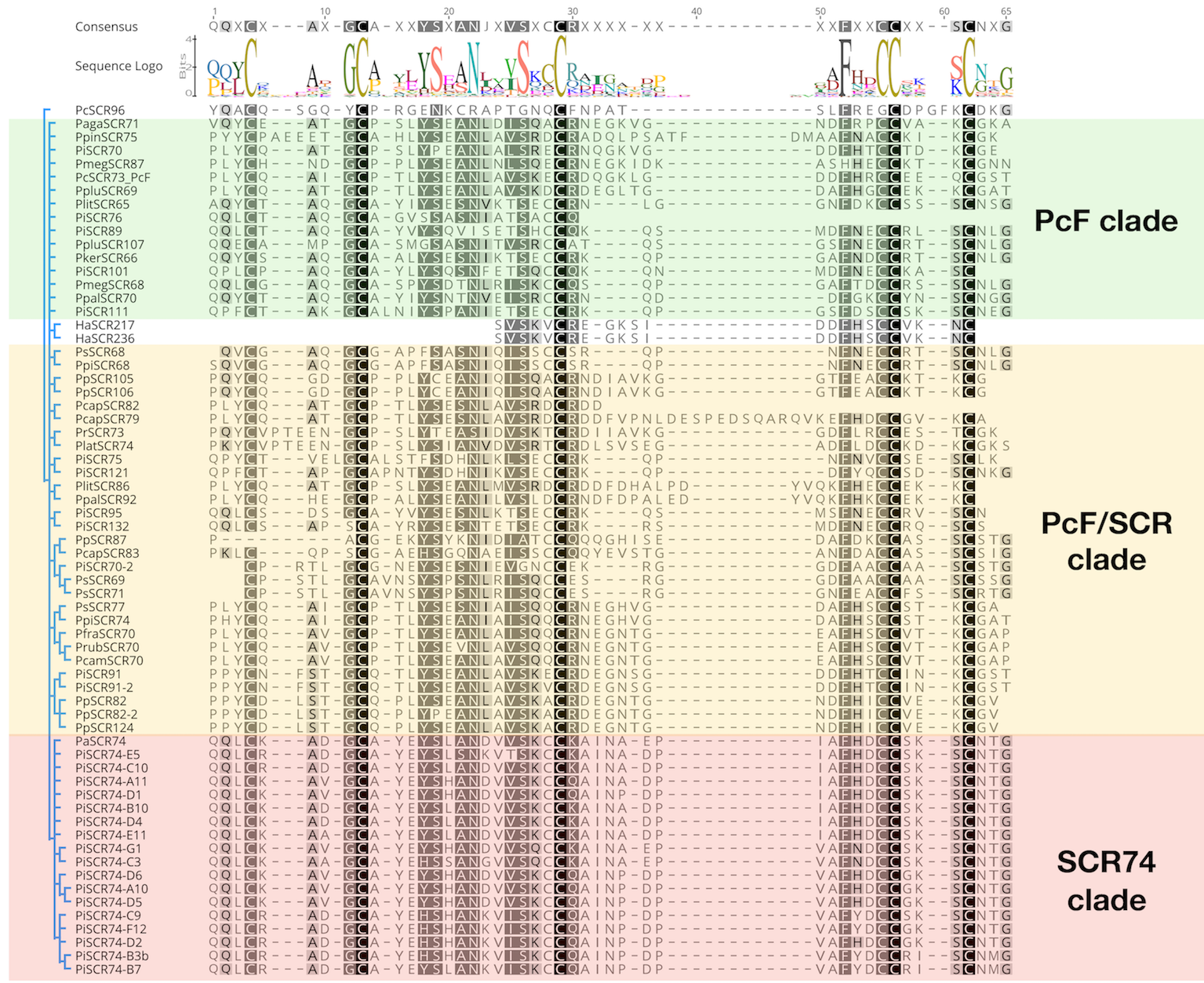

Supplement: FIG S1 [file mBio.00947-20-sf001.tif]

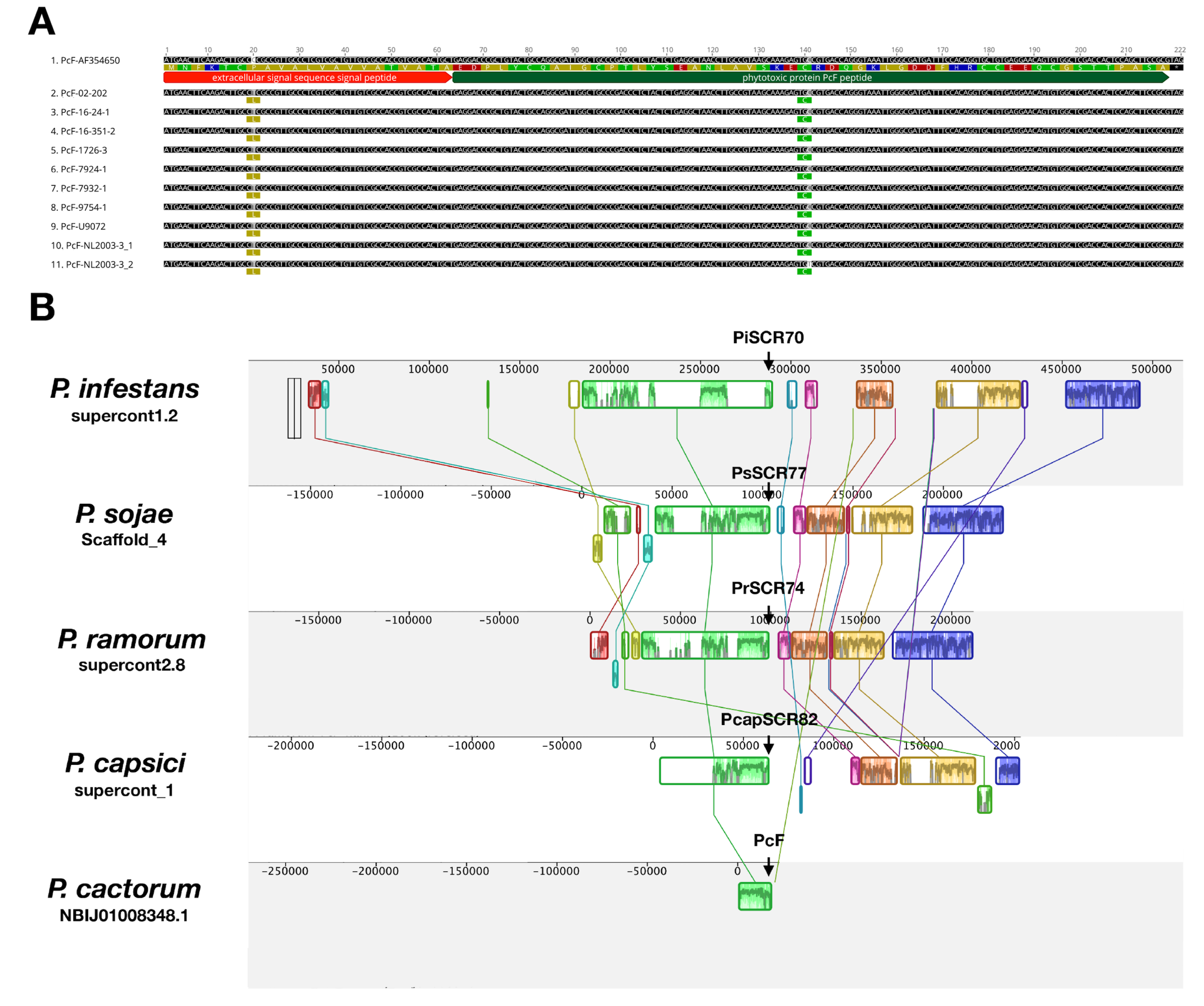

Supplement: FIG S2 [file mBio.00947-20-sf002.tif]

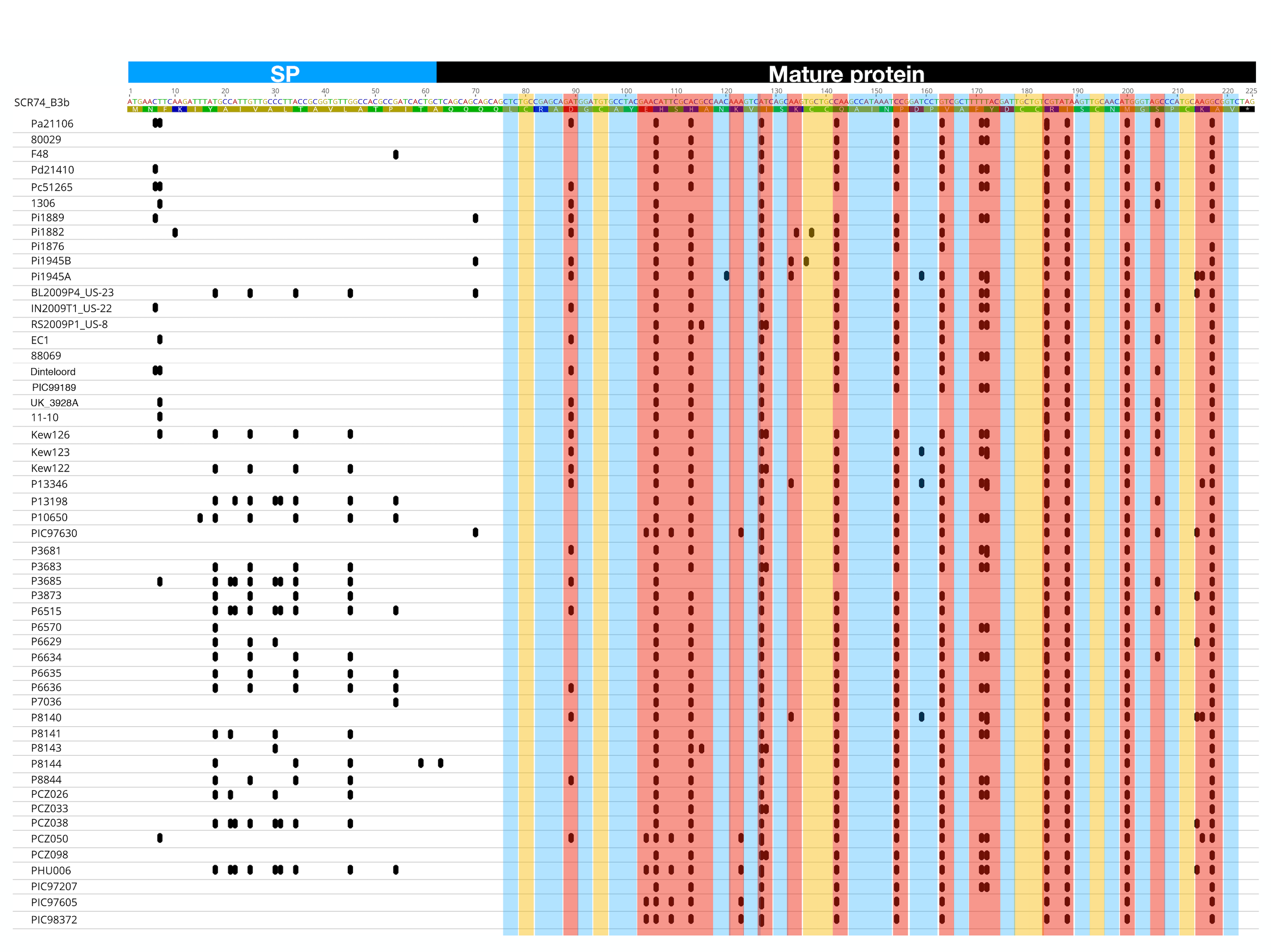

Supplement: FIG S3 [file mBio.00947-20-sf003.tif]

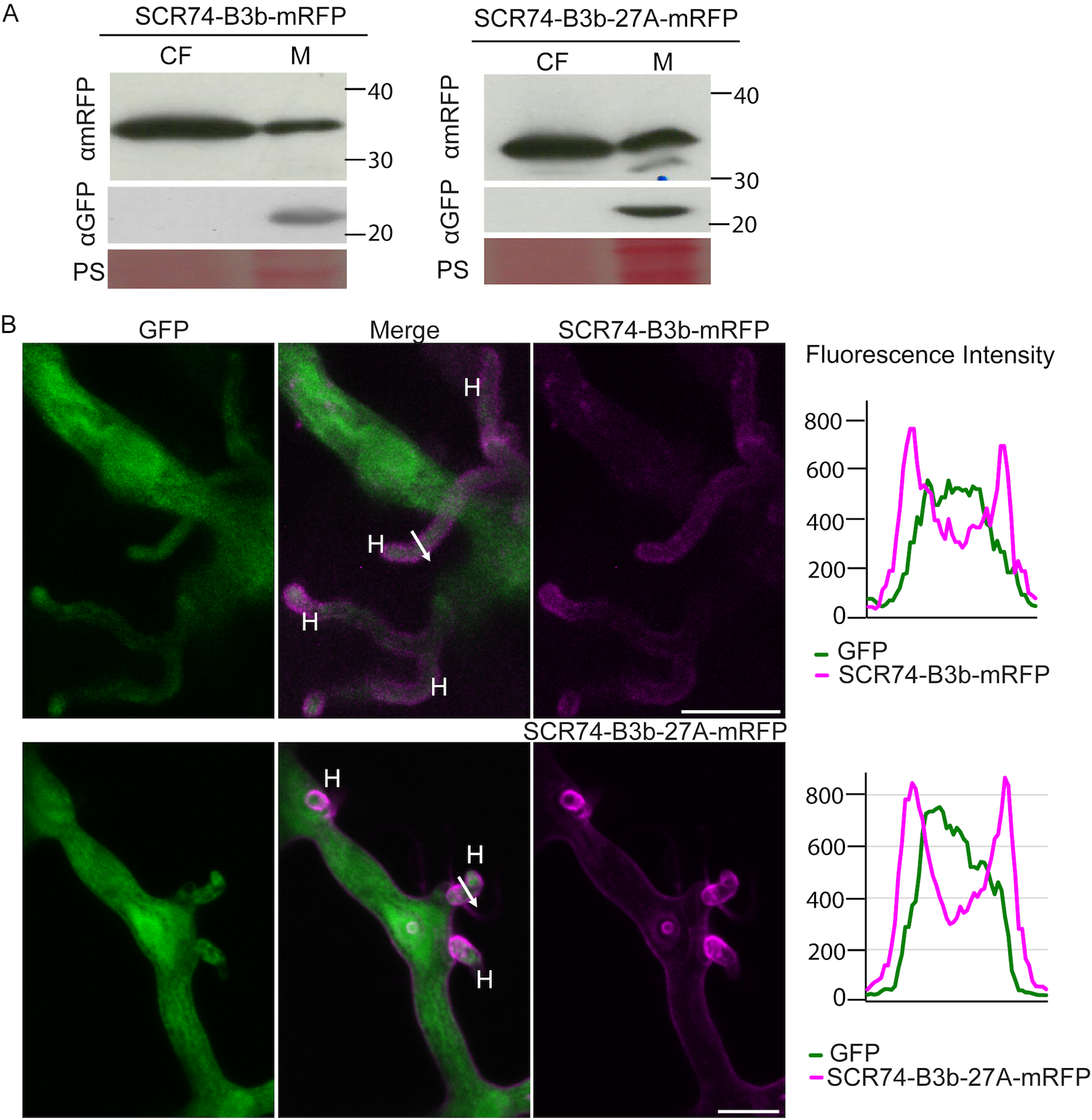

Supplement: FIG S4 [file mBio.00947-20-sf004.tif]

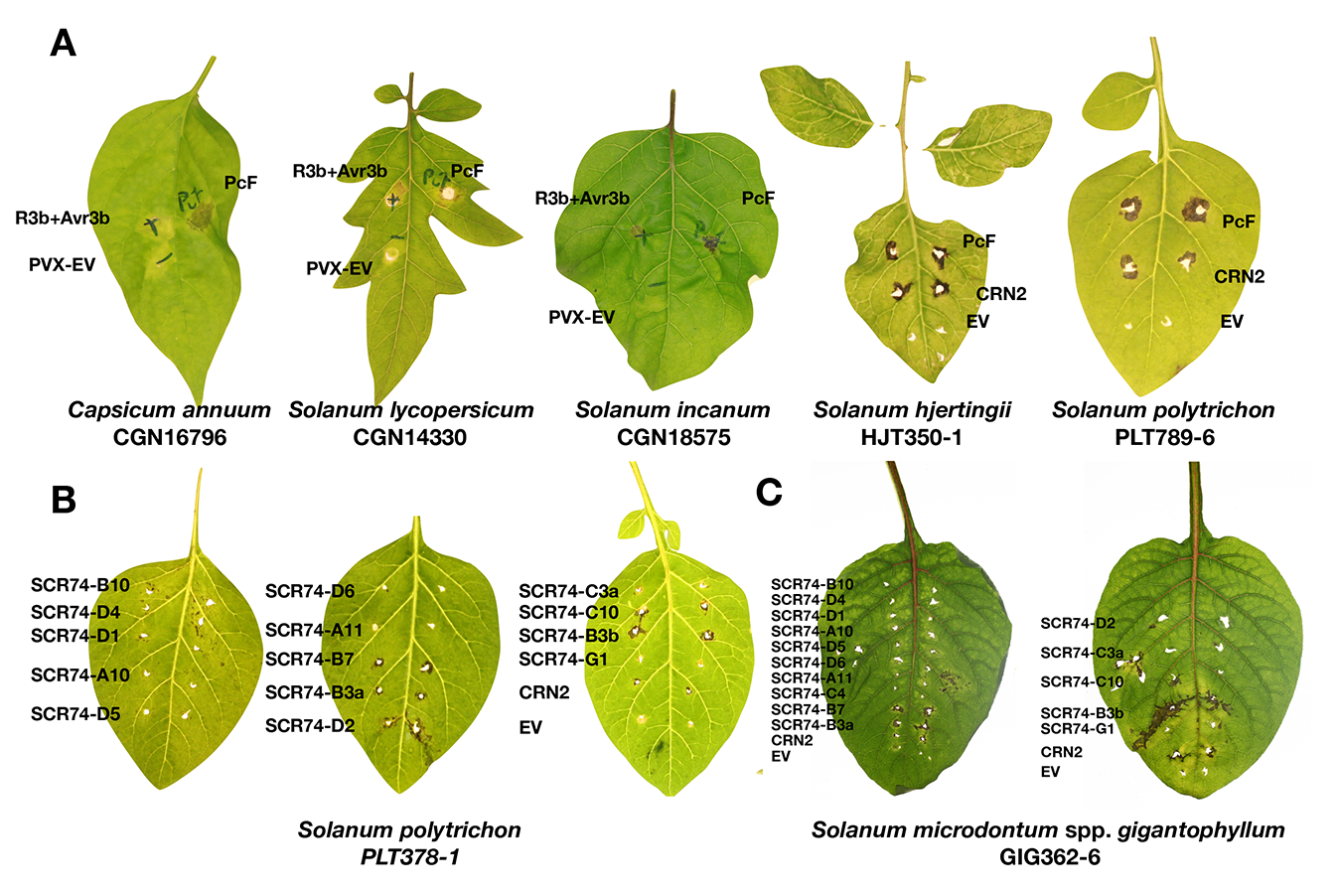

Supplement: FIG S5 [file mBio.00947-20-sf005.tif]

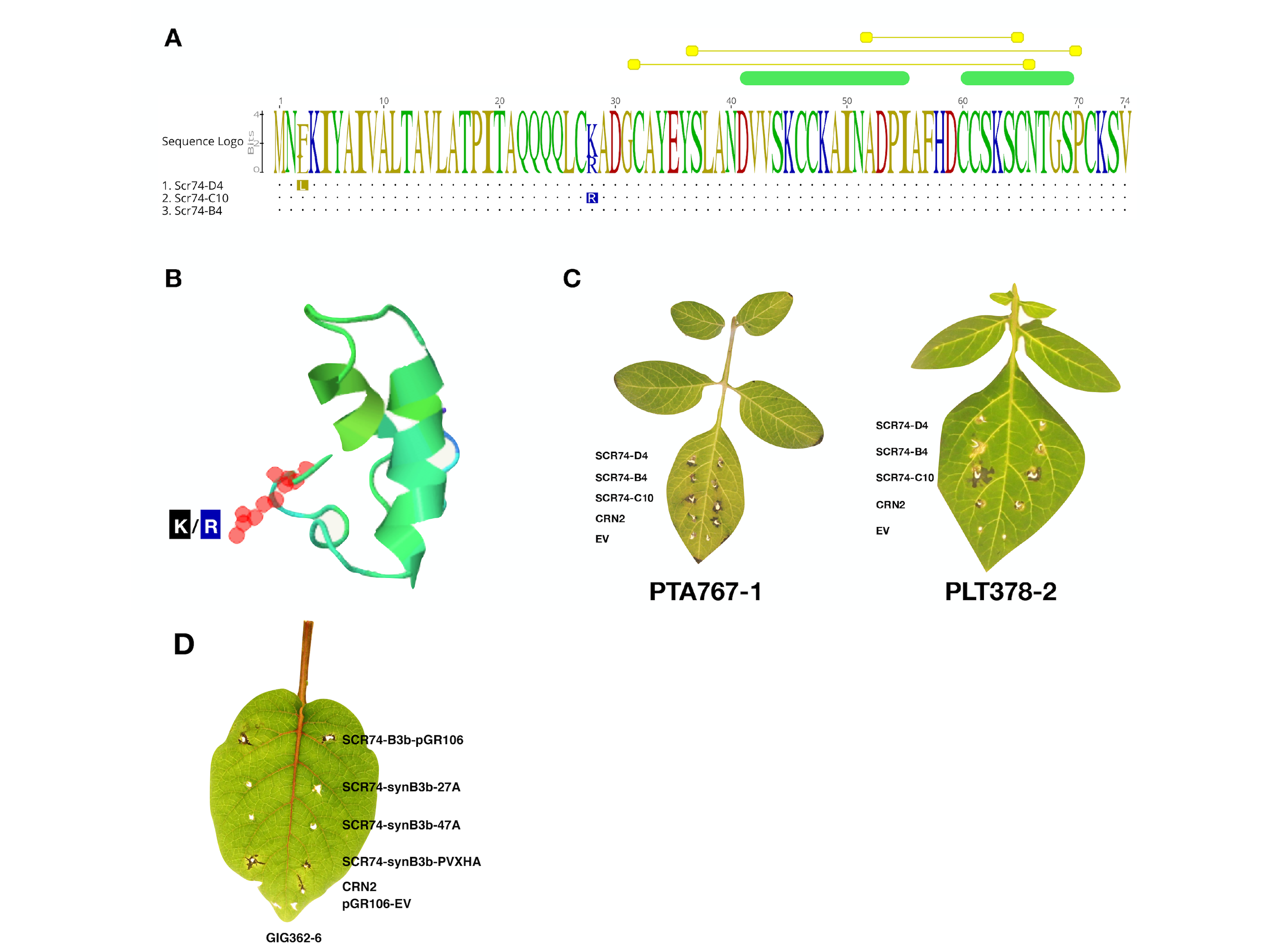

Supplement: FIG S6 [file mBio.00947-20-sf006.tif]

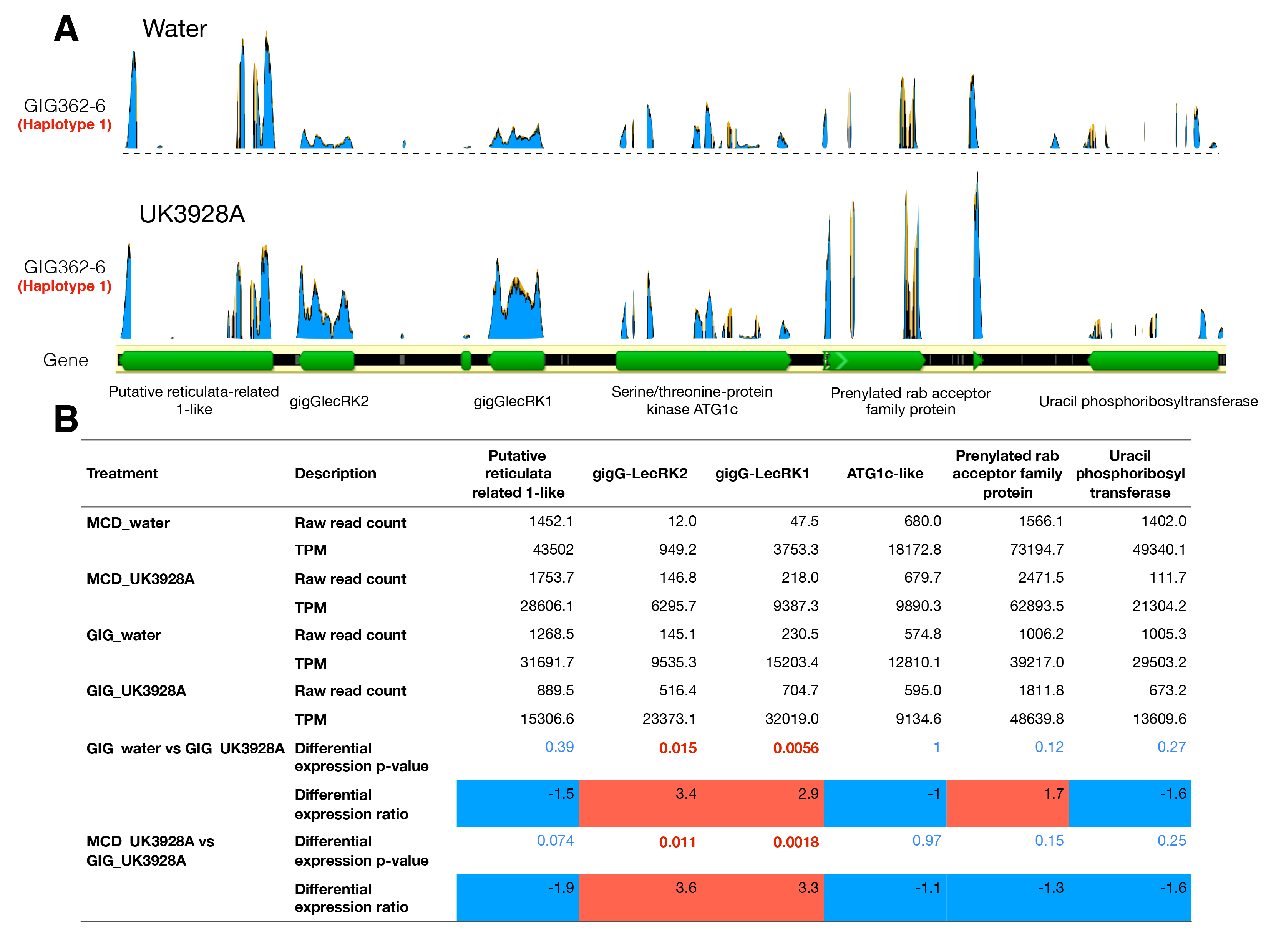

Supplement: FIG S7 [file mBio.00947-20-sf007.tif]
