# Supplementary material for: Divergent Evolution of PcF/SCR74 Effectors in Oomycetes Is Associated with Distinct Recognition Patterns in Solanaceous Plants
Source: mBio. 2020 Jun 30;11(3):e00947-20. doi: 10.1128/mBio.00947-20 (PMC7327169; doi:10.1128/mBio.00947-20)
Supplement: TEXT S1 [file mBio.00947-20-s0001.docx]

**SI Materials and Methods**

## **Phylogenetic analysis of PcF/SCR proteins**

45 PcF domain-containing proteins (IPR018570) were obtained from InterPro (<https://www.ebi.ac.uk/interpro/)>. The protein sequences were aligned by MAFFT v7.309 (38) and Geneious R10 ([http://www.geneious.com](http://www.geneious.com/)) (45). Redundant sequences were removed manually based on the alignment outputs. Additionally, 19 publicly available *Phytophthora* genomes were searched for *Scr74* or *PcF* homologs by tBlastn, the genomes including *P. infestans*, *P. parasitica*, *P. cacotrum*, *P. capsica*, *P. sojae*, *P. ramorum*, *P. agathidicida*, *P. cambivora*, *P. fragariae*, *P. keernoviae*, *P. lateralis*, *P. litchi*, *P. palmivora*, *P. pinifolia*, *P. pisi*, *P. pluvialis*, *P. rubi* and *P. megakarya*, as well as *Hyaloperonospora arabidopsidis*. In total, 65 unique PcF/SCR proteins were identified and used in this study. A Neighbor-Joining tree was performed by Geneious R10, using Jukes-Cantor model. The phylogeny network was made by SplitTree4 (39), computed by the NeighborNet method and the tree was displayed using the EqualAngle algorithm. The proportion of different positions between two sequences were computed by UncorrectedP.

## ***P. infestans* sequencing data from GenBank**

57 publicly available sequencing data of *P. infestans* were used for studying the genetic diversity of SCR74 in *P. infestans*. These data include some historical samples like Pi1845A, Pi1845B, Pi1882, Pi1876, Pi1889 (46) and some hybrids *P. andiana* EC3394, PaX and P13803 (47). SCR74-B3b (GenBank: AY723717.1) was used as query for obtaining the reads based on each sequence read archive (SRA) database from NCBI. The SAM format outputs were imported into Geneious R10, SCR74-B3b was used as reference sequence. Then the Find Variation/SNPs function from Geneious R10 were used for detecting the SNPs from each individual isolate.

***Phytophthora* isolates and DNA isolation of *Phytophthora cactorum***

*Phytophthora cactorum* isolates from USA or Europe used in this study were listed in Table S1.

The isolates were routinely cultured on V8 medium and stored at 20°C in the dark. One liter of V8 medium contains 200mL of V8-vegetable juice, 800mL tap water, 20g of agarose (n°3) and 3g of CaCO3. Genomic DNA was extracted from *Phytophthora cactorum* mycelia by DNeasy Plant Mini Kit (QIAGEN). The *PcF* genes were amplified by primer PcF-F: ATGAACTTCAAGACTTGCC and PcF-R: AAACAGTCTACGCGGAAG with Phusion PCR high-fidelity DNA polymerase. After A-tailing by DreamTaq DNA polymerase, the *PcF* genes were ligated into pGEM-T Easy Vector system (Promega) and transformed to *E. coli* DH5α competent cells. The plasmid of positive clones were isolated by QIAprep Spin Miniprep Kit (QIAprep® QIAGEN), then sent for sequencing (GATC). The sequence analysis and alignment were performed by Geneious R10.

## **Plant materials**

The potatoes were propagated in vitro, they were grown for 2 weeks in a climate room at 22°C before moving to the greenhouse. They were further grown for 4-6 weeks in the greenhouse before the PVX agroinfection.

## **PVX agro-infection**

The 13 SCR74 homologs (10) were cloned into PVX vector pGR106, PcF, PsSCR68 and PcSCR96 were synthesized by Genewiz (https://www.genewiz.com/) and cloned into a modified gateway compatible PGWC-PVX vector. The effectors were then transformed into *Agrobacterium tumefaciens* strain GV3101. Before PVX agro-infection, the *Agrobacterium* carrying the target effectors were grown 2 days in liquid LB median, then 2 days in solid LB median. The *Agrobacterium* then were collected by sterilized spreading rod and toothpicks were used for the agro-infection (48).

## **Mutagenesis**

Two SCR74-B3b cysteine mutations (SCR74-B3b-27A and SCR74-B3b-47A) together with the wild type SCR74-B3b were codon optimized and synthesized by Genewiz and cloned into PGWC-PVX.

## **Immunoblotting**

*Phytophthora infestans* transformants expressing either SCR74-B3b-mRFP or SCR74-B3b-27C-mRFP were cultured in amended lima bean (ALB) liquid medium (49). *In vitro* grown mycelium was harvested by centrifugation at 2 dpi, then transferred into 1 ml of ALB liquid medium. The culture filtrate (CF) was retained separately after 24 h incubation, and four times the sample volume of cold (-20°C) acetone (Thermo Fisher Scientific, Loughborough, UK) was used to precipitate proteins overnight. Protein was precipitated at 10,000 g for 10 min (28). *P. infestans* mycelium and resuspended CF were mixed in 2x SDS PAGE loading buffer (100 mM Tris-HCl (pH 6.8), 200 mM DTT, 4 % (w/v) SDS, 0.2 %(w/v) Bromophenol Blue, 20 % (v/v) glycerol). Samples (10 µl) were loaded onto a 10 % Bis-Tris SDS PAGE gel. The gel was run with 1X MES SDS running buffer (Invitrogen) for 30min at 80 V, then at 110 V for another 1 h. Gel blotting and membrane blocking were carried out as described by (50). amRFP and aGFP primary antibodies (Sigma-Aldrich) were used at 1 : 4000 and 1 : 2000 dilution, respectively. Secondary antibodies anti-rat immunoglobulin G (IgG) horseradish peroxidase (HRP) or anti-rabbit IgG HRP (Sigma-Aldrich) were used at 1: 5000 dilutions. Protein bands imaged with Amersham Hyperfilm^TM^ ECL, were processed with an Xograph imaging system.

## **Confocal imaging**

*Nicotiana benthamiana* leaf tissues were mounted on slides and imaged on Nikon A1R confocal microscope. GFP was imaged with 488 nm excitation and emissions collected between 500 and 530 nm, respectively. mRFP fluorescent proteins were excited with 561 nm light and fluorophores emission detected between 600 and 630 nm. Pinhole was set to 1 Airy unit for the longest wavelength of fluorophore. Cells expressing low levels of fluorescence were imaged to minimize over-expression artefacts. Projections were collected from leaf tissue infected by *P. infestans* transformants, the leaf tissue was not heavily inoculated for minimizing auto-fluorescence from the cell damage and death. Images were processed with propriety confocal software. Figures were analyzed with Adobe Photoshop and Adobe Illustrator.

## **Generating a BAC library of the genotype GIG362-6 and isolation of BAC clones covering the mapping interval**

The BAC library of GIG362-6 was generated by Bio S&T (Canada). The BAC clones were stored in three 96 deep well plates, with 490 clones in each well, the total number of clones was 141,120. The average insert size was 151kb. The coverage was >11x (GIG362-6 is a highly heterozygous diploid potato, the genome size is around 2 x 900Mb). The BAC construction vector was pCC1BAC (HindIII) and the *E. coli* strain was DH 10B. The BAC library was stored at -80°C.

The BAC DNA was isolated by QIAprep miniprep (Qiagen) kit with minor revision. After adding the P3 step in the industrial instruction, the tubes were closed with caps and mixed by inverting, and spin down for 10 minutes, the supernatant was transferred to fresh tubes and 0.6 V isopropanol was added, then mixed and centrifuged for 10 min. The pellets were washed with 500 µL of 70% cold ethanol, centrifuged for 5 minutes. The pellets were air dried and re-suspended in 50 µL MQ.

For BAC clone isolation, the markers in the mapping interval were firstly tested on BAC DNA and positive pools with 490 clones were detected. Then the selected pools were diluted to ≈1 x 10^-6^ – 1 x 10^-8^ with sterilized water. The diluted cultures should contain around 1000 clones/ ml. Then 1ml culture was plated on 1 square LB plate with 25 mg/L chloromycetin and grown overnight at 37°C. Next day, 1152-2304 single colonies were picked up into 384 plates with liquid LB (25 mg/L chloromycetin) and grown overnight at 37°C. Then the 384 plates were pooled into 96 well plates for PCR. If a positive well was found in the 96 well plates, another PCR was performed to obtain single BAC clones.

The single BAC clones were further tested on solid LB plates, 5 single colonies were tested by PCR. The BAC clones were sequenced by French Plant Genomic Resource Center (INRA-CNRGV) using PacBio technology and the BAC clones were characterized by estimating their insert size and BAC ends by Sanger sequencing (BES). Finally, PacBio RS libraries with 8-12 kb insert size (based on a minimum of 60X with average reads length up to 5 kb) were produced.

**RNAseq of GIG362-6 and MCD360-1**

Detached leaves of GIG362-6 and MCD360-1 were treated by water control and a zoospore suspension (50.000 spores/l) of *P. infestans* isolate UK3928A. After 48 hours, leaf disks were sampled into 2 ml RNase free tubes and frozen by liquid nitrogen. The four samples were grinded by TissueLyser II (QIAGEN). Then 100mg samples were used for RNA isolation by RNeasy Plus Mini Kit (QIAGEN) following industrial instructions. The gDNA eliminator spin column from the kit could efficiently remove the gDNA. The four RNA samples were tested by agarose electrophoresis, quantified by Nanodrop (ThermoFisher) and send to the Genome Analysis Centre (TGAC, UK) with dry ice for RNA sequencing. The samples were sequenced by HiSeq2000 with a 100bp paired end read metric.

The raw reads were first checked with FastQC (v0.10.0; http://www.bioinformatics.babraham.ac.uk/projects/fastqc/) and the adapters were trimmed with trimmomatic v0.36 (51). The trimmed reads were mapped to the BAC sequences using STAR v2.5 (52). The output BAC files were imported to Geneious R10, the RPKM (Reads Per Kilobase Million), FPKM (Fragments Per Kilobase Million) and TPM (Transcripts Per Kilobase Million) were calculated in Geneious R10. The differential expression levels were also calculated in Geneious R10, the transcripts were compared and normalized by median of gene expression ratios.

**References**

**Bolger, A.M., Lohse, M. and Usadel, B.** (2014) Trimmomatic: a flexible trimmer for Illumina sequence data. *Bioinformatics*, **30**, 2114–2120.

**Bruck, R.I., Fry, W.E., Apple, A.E. and Mundt, C.** (1981) Effect of protectant fungicides on the developmental stages of Phytophthora infestans in potato foliage. *Phytopathology*.

**Dobin, A., Davis, C.A., Schlesinger, F., Drenkow, J., Zaleski, C., Jha, S., Batut, P., Chaisson, M. and Gingeras, T.R.** (2013) STAR: ultrafast universal RNA-seq aligner. *Bioinformatics*, **29**, 15–21.

**Du, J., Rietman, H. and Vleeshouwers, V.G.A.A.** (2014) Agroinfiltration and PVX agroinfection in potato and *Nicotiana benthamiana*. *JoVE*, e50971–e50971.

**Huson, D.H. and Bryant, D.** (2006) Application of phylogenetic networks in evolutionary studies. *Mol Biol Evol*, **23**, 254–267.

**Katoh, K. and Standley, D.M.** (2013) MAFFT multiple sequence alignment software version 7: improvements in performance and usability. *Mol Biol Evol*, **30**, 772–780.

**Kearse, M., Moir, R., Wilson, A., et al.** (2012) Geneious Basic: An integrated and extendable desktop software platform for the organization and analysis of sequence data. *Bioinformatics*, **28**, 1647–1649.

**Liu, Z., Bos, J.I.B., Armstrong, M., et al.** (2005) Patterns of diversifying selection in the phytotoxin-like *scr74* gene family of *Phytophthora infestans*. *Mol Biol Evol*, **22**, 659–672.

**Martin, M.D., Cappellini, E., Samaniego, J.A., et al.** (2013) Reconstructing genome evolution in historic samples of the Irish potato famine pathogen. *Nat Commun*, **4**, 2172.

**Martin, M.D., Vieira, F.G., HO, S.Y.W., Wales, N., Schubert, M., Seguin-Orlando, A., Ristaino, J.B. and Gilbert, M.T.P.** (2016) Genomic Characterization of a South American *Phytophthora* Hybrid Mandates Reassessment of the Geographic Origins of *Phytophthora infestans*. *Mol Biol Evol*, **33**, 478–491.

**McLellan, H., Boevink, P.C., Armstrong, M.R., Pritchard, L., Gomez, S., Morales, J., Whisson, S.C., Beynon, J.L. and Birch, P.R.J.** (2013) An RxLR Effector from *Phytophthora infestans* Prevents Re-localisation of Two Plant NAC Transcription Factors from the Endoplasmic Reticulum to the Nucleus B. Tyler, ed. *PLoS Pathog*, **9**, e1003670.

**Wang, S., Boevink, P.C., Welsh, L., Zhang, R., Whisson, S.C. and Birch, P.R.J.** (2017) Delivery of cytoplasmic and apoplastic effectors from *Phytophthora infestans* haustoria by distinct secretion pathways. *New Phytol*, **42**, 385.
